# STAT3 inhibition eradicates multiple myeloma stem-like cells and induces immunogenic cell death

**DOI:** 10.1097/BS9.0000000000000301

**Published:** 2026-07-31

**Authors:** Yuqian Sha, Wenyu Li, Yan Zhang, Tengyuan Liu, Mei Yuan, Wenya Wang, Jianquan Gu, Hai Cheng, Mingshan Niu, Peiyu Yang, Yao Yao

**Affiliations:** a Blood Disease Institute, Key Laboratory of Bone Marrow Stem Cell, Xuzhou Medical University, Xuzhou, China; b Department of Hematology, The Affiliated Hospital of Xuzhou Medical University, Xuzhou, China; c Tianjin University of Science and Technology, Tianjin, China; d Obstetrics and Gynecology, The Affiliated Hospital of Xuzhou Medical University, Xuzhou, China

**Keywords:** Cancer stem cells, ICD, Napabucasin, STAT3

## Abstract

Signal transducer and activator of transcription 3 (STAT3) is a pivotal oncogenic driver in multiple myeloma (MM), and its constitutive activation promotes malignant plasma cell proliferation, survival, and drug resistance in the bone marrow microenvironment. Despite therapeutic advances, MM remains incurable due to persistent STAT3-driven tumorigenesis and the resilience of MM stem cells. We investigated the therapeutic potential of napabucasin (BBI608), a novel STAT3 inhibitor, in MM. Our data demonstrated that BBI608 potently suppressed MM cell proliferation in vitro and in vivo, while significantly impairing the clonogenic potential and inducing robust apoptosis. Mechanistically, BBI608 exhibited dual efficacy by targeting bulk tumor cells and eradicating the stem-like compartment of MM cells, thereby addressing a critical therapeutic challenge. Moreover, we revealed that BBI608 triggered immunogenic cell death (ICD) via the activation of endoplasmic reticulum (ER) stress and the unfolded protein response (UPR), which subsequently enhanced T-cell–mediated anti-tumor immunity. Our findings highlight STAT3 inhibition as a promising strategy to simultaneously eradicate MM cells, target stem cell reservoirs, and harness anti-tumor immunity, providing a robust rationale for the clinical translation of BBI608 in MM therapy.

## 1. INTRODUCTION

Accumulating evidence indicates that the constitutive activation of signal transducer and activator of transcription 3 (STAT3) in tumor cells contributes to the progression of various cancers, including multiple myeloma (MM). Persistent activation of STAT3 plays a critical role in multiple aspects of MM pathogenesis, such as cell survival, proliferation, antiapoptotic mechanisms, bone marrow (BM) microenvironment modulation, and immune evasion. Moreover, STAT3 overexpression in MM has been associated with a poor prognosis and may significantly contribute to microenvironment-mediated treatment resistance.^1–3^

Cancer stem cells (CSCs) represent a rare subpopulation of tumors and are pivotal in driving resistance to chemotherapeutic agents and cancer recurrence. Since the first prospective identification of CSCs in solid tumors, the CSC hypothesis has emerged as an increasingly prominent research focus.^4^ Although the existence and identification of MM stem cells remain controversial, current data suggest that the MM cell population contains poorly differentiated progenitor cells exhibiting CSC characteristics, known as multiple myeloma stem-like cells (MMSCs).^5,6^ These cells are characterized by their ability to maintain the drug resistance associated with MM relapse.^7,8^ STAT3 plays a key role in maintaining the expression of genes that are important for stem cell phenotypes and can be used as a biomarker for CSCs.^9^

Moreover, the immune system is compromised in patients with MM. Although targeted immunotherapeutic interventions have shown promising efficacy and hold great clinical potential, challenges, such as relapse and drug resistance, remain. Immunogenic cell death (ICD) is a distinct form of regulated cell death that serves as a potent immunotherapeutic strategy for hematologic malignancies.^10^ Dying tumor cells release a set of danger signals known as damage-associated molecular patterns (DAMPs), which facilitate their recognition by dendritic cells (DCs), promote tumor antigen processing and presentation to CD4+ and CD8+ T cells, and ultimately stimulate an anti-tumor immune response.^11,12^ Activation of endoplasmic reticulum (ER) stress, particularly through the unfolded protein response (UPR), is a crucial driver of ICD. The protein kinase R-like endoplasmic reticulum kinase (PERK)-mediated arm of the UPR is essential for ICD induction, and MM cells, which rely heavily on UPR signaling for survival, are particularly susceptible to this process.^10^ To date, only a small subset of anticancer agents have been identified as bona fide ICD inducers, including bortezomib, a standard MM therapy that is an effective ICD inducer in myeloma cells.^13^

BBI608 is a small-molecule inhibitor that targets STAT3, a master regulator of stemness that induces apoptosis and disrupts the self-renewal in CSCs.^14,15^ In this study, we investigated the therapeutic potential of STAT3 pathway inhibition in MM by examining its effects on bulk MM cell proliferation and stem cell expansion using BBI608. Furthermore, we explored the effects of STAT3 blockade on ICD induction in MM cells. These findings establish a strong preclinical rationale for the clinical translation of STAT3-targeted therapy for MM treatment.

## 2. MATERIALS AND METHODS

### 2.1. Cell lines and patient samples

The RPMI 8226, IM-9, U266, and OPM-2 cell lines were purchased from the American Type Culture Collection (ATCC). The KMS-11 cell line was cultured and maintained in the laboratory. These cells were grown in RPMI-1640 (KeyGEN Biotech, Nanjing, China) supplemented with 10% fetal bovine serum (BC-SE-FBS01; Bio Channel, Nanjing, China), 100 mg/L penicillin-streptomycin, and 2 mM L-glutamine. The human BM stromal cell line HS-5 was obtained from ATCC and cultured in Dulbecco’s Modified Eagle Medium (DMEM) (KeyGEN Biotech, China). All cells were cultured in a humidified incubator at 37°C with 5% CO_2_. Mononuclear cells were isolated from the BM aspirates of 17 patients with MM and umbilical cord blood from 7 healthy donors using Ficoll-Paque PREMIUM density gradient separation medium, washed twice with sterile phosphate-buffered saline (PBS), and cultured in 1640 medium containing 10% fetal bovine serum. This study was approved by the Medical Ethics Committee of the Affiliated Hospital of Xuzhou Medical University.

### 2.2. Reagents and antibodies

BBI608 was purchased from Topscience (Shanghai, China), dissolved in dimethyl sulfoxide (DMSO) at a stock concentration of 50 mM, and stored at −20°C. The cell counting kit-8 (CCK-8) was purchased from Vicmed (Xuzhou, China), and the apoptosis assay kit was purchased from eBioscience, an Affymetrix Company (Shanghai, China). Polyclonal antibodies against p21, p27, p53, Bax, Bcl-2, GAPDH, β-tubulin, PARP, caspase-3, cleaved caspase-3, STAT3, and p-STAT3 were purchased from Cell Signaling Technology (Beverly, Massachusetts). Anti-EIF2α, p-EIF2α, PERK, p-PERK, PKR, p-PKR antibodies were purchased from Proteintech (Wuhan, China). Recombinant human macrophage colony-stimulating factor (300-25), recombinant human soluble receptor activator of nuclear factor kappa-B (NF-κB) ligand (RANKL; 500-P133), recombinant human interleukin-4 (IL-4; #200-04), and recombinant human granulocyte-macrophage colony-stimulating factor (GM-CSF; #300-03) were purchased from PeproTech (Hamburg, Germany). The tartrate-resistant acid phosphatase (TRACP) and ALP double-staining kit (MK300) was purchased from TaKaRa (Beijing, China).

### 2.3. Extraction of peripheral blood mononuclear cells and TRAP staining of osteoclasts

Mononuclear cells were isolated from the peripheral blood mononuclear cells (PBMCs) of normal volunteers. The culture medium was supplemented with 25 ng/mL of M-CSF and 50 ng/mL of RANKL and refreshed 3 times a week. BBI608 was added at 200 and 400 nM. After 2 weeks of culture, the cells were fixed and stained for TRACP using the TRACP & ALP double-staining kit, according to the manufacturer’s instructions. Osteoclasts were defined as TRAP+ cells containing 3 or more nuclei. Each osteoclast formation assay was performed at least 3 times using PBMCs from different donors.

### 2.4. Isolation of side population cells

KMS-11 cells were cultured in RPMI-1640 containing 2% FBS and treated with 5 μg/mL Hoechst 33342 (Life Technologies, Carlsbad, California) in the presence or absence of designated doses of BBI608. The cells were incubated at 37°C for 90 minutes. After incubation, the cells were washed 3 times with ice-cold 1× PBS. To distinguish dead cells, propidium iodide (PI; 2 μg/mL; Sigma, St. Louis, Missouri) was added immediately before analysis. Side population (SP) cells were isolated using a BD Influx Cell Sorter (BD, Franklin Lakes, New Jersey).

### 2.5. Quantitative real-time polymerase chain reaction

MM cells were lysed with 1 mL TRIzol reagent (Invitrogen, Carlsbad, California) to obtain RNA, and cDNA was synthesized with the PrimeScript RT Reagent Kit (TaKaRa, Shiga, Japan). The quantitative real-time polymerase chain reaction (qRT-PCR) reaction was performed on a LightCycler 480 II Real-Time PCR System (Roche, Basel, Switzerland) with a total reaction volume of 20 μL. The amplification program was pre-denaturation at 95°C for 30 seconds, followed by 40 cycles of amplification. Each cycle consisted of denaturation at 95°C for 10 seconds and annealing/extension at 60°C for 30 seconds. SYBR Green fluorescence signals were collected at the end of the 60°C step (Vazyme, Cat. No. Q312-02).

qRT-PCR was performed using the LightCycler^®^ 480 SYBR Green I Master (Roche, Basel, Switzerland). The following primers were used:

GAPDH, forward 5′-GCACCGTCAAGGCTGAGAAC-3′

and reverse 5′-TGGTGAAGACGCCAGTGGA-3′;

NANOG, forward 5′-CAATGGTGTGACGCAGGGA-3′

and reverse 5′-TGCACCAGGTCTGAGTGTTC-3′;

OCT4, forward 5′-ACATCAAAGCTCTGCAGAAAGAACT-3′

and reverse 5′-CTGAATACCTTCCCAAATAGAACCC-3′;

SOX2, forward 5′-AACCAGCGCATGGACAGTTA-3′

and reverse 5′-GACTTGACCACCGAACCCAT-3′;

LIN28A, forward 5′-AAGGAGACAGGTGCTACAAC-3′

and reverse 5′-AATAGCCCCCACCCATTGTG-3′.

### 2.6. RNA library preparation and sequencing

KMS-11 and RPMI 8226 cells were treated with BBI608 (0, 1000, and 1500 nM) for 24 hours. Total RNA was extracted, and mRNA was enriched using oligo(dT)-attached magnetic beads. Libraries were constructed using an Optimal Dual-mode mRNA Library Prep Kit (BGI, Shenzhen, China). Briefly, mRNA was fragmented, and first- and second-strand cDNA were synthesized. After end repair and A-tailing, adaptors were ligated, followed by PCR amplification. Single-stranded circular libraries were generated and amplified using rolling circle amplification to form DNA nanoballs. Sequencing was performed using the BGI G400/T7/T10 platform to generate paired-end 100 or 150 bp reads. The RNA-seq data were deposited in the Gene Expression Omnibus (GEO) database under accession number GSE299035. Gene set enrichment analysis (GSEA) was performed using the GSEA software (v4.3.2, Broad Institute) with the Hallmark gene sets (h.all.v2023.1) from the Molecular Signatures Database (MSigDB).

### 2.7. ICD-related molecules analysis

The cells were treated with BBI608 for 24 hours and harvested to detect ICD-related indicators. Briefly, for the analysis of calreticulin (CRT) exposure, the cells were stained with Alexa Fluor 647-conjugated anti-calreticulin antibody (Abcam, Cambridge, UK) and PI (eBioscience, San Diego, CA) on ice for 30 minutes and detected by flow cytometry. The fluorescence intensity was assessed on viable (PI-negative) cells. For adenosine triphosphate (ATP) release, an ATP Assay Kit (Beyotime, Shanghai, China) was used for the quantitative analysis of ATP, in accordance with the manufacturer’s instructions. For heat shock protein 70/90 (HSP70/90) secretion assays, the cells were stained with an anti-HSP70/90 primary antibody and CoraLite488-conjugated goat anti-rabbit immunoglobulin G (Proteintech, Wuhan, China). Cell surface HSP70/90 expression was measured using flow cytometry.

### 2.8. DC culture and maturation assay

Monocyte-derived DCs isolated from PBMCs of healthy donors were cultured in 100 mm culture dishes for 3 hours. Subsequently, the suspended cells were removed, and the adherent cells were placed in complete RMIP 1640 medium containing 100 ng/mL granulocyte-macrophage colony-stimulating factor (PeproTech, Cranbury, NJ) and 100 ng/mL IL-4 (PrepoTech) and cultured for 5 days to generate immature DCs. Subsequently, the immature DCs were cultured alone or cocultured with untreated or BBI608-pretreated MM cells for 48 hours. Cells labeled with CD45-APC, CD11c-BV421, CD80-FITC, and CD86-PE antibodies (BioLegend, San Diego, CA) were detected by flow cytometry, and the expression levels of CD80 and CD86 on CD11c+ cells were evaluated.

### 2.9. T-cell isolation and T-cell–mediated cytotoxicity assay

T cells were isolated from PBMCs using the EasySep™ Human T-Cell Isolation Kit (STEMCELL), following the manufacturer’s protocol. Subsequently, the isolated T cells were cultured in RPMI-1640 medium supplemented with 10% FBS until immature DCs were generated. For the T-cell–mediated cytotoxicity assay, MM cells were treated with BBI608 for 24 hours and labeled with carboxyfluorescein diacetate succinimidyl ester (CFSE; BD Biosciences). The labeled MM cells were then cocultured with immature DCs and T cells at the indicated ratios for 48 hours. The percentage of viable MM cells was determined using flow cytometry.

### 2.10. MM mouse model

The mouse model of MM was established using NOD.Cg-Prkdc^scid^Il2rg^tm1Wjl^ (NSG) mice (6–8 weeks old, male) were procured from Beijing Vital River Laboratories. The animal experimental protocol was reviewed and approved by the Ethics Committee of Xuzhou Medical University. The NSG mice were intravenously injected with 1 × 10^6^ RPMI 8226 cells suspended in 100 μL of PBS. The mice were subsequently treated with either a placebo or BBI608 (30 mg/kg) on a 5-day-per-week schedule. Upon the onset of paralysis in the mice, euthanasia was performed according to the ethical guidelines.

### 2.11. Statistical analysis

All results were given as the mean ± standard deviation (SD) of at least 2 independent experiments, and significant differences among the 3 groups were calculated using one-way analysis of variance (ANOVA). Significant differences between the 2 groups were assessed using the Student *t* test. *p* < 0.050 was considered statistically significant.

## 3. RESULTS

### 3.1. BBI608 blocked myeloma cell growth in vitro and in vivo

**Figure 1.**
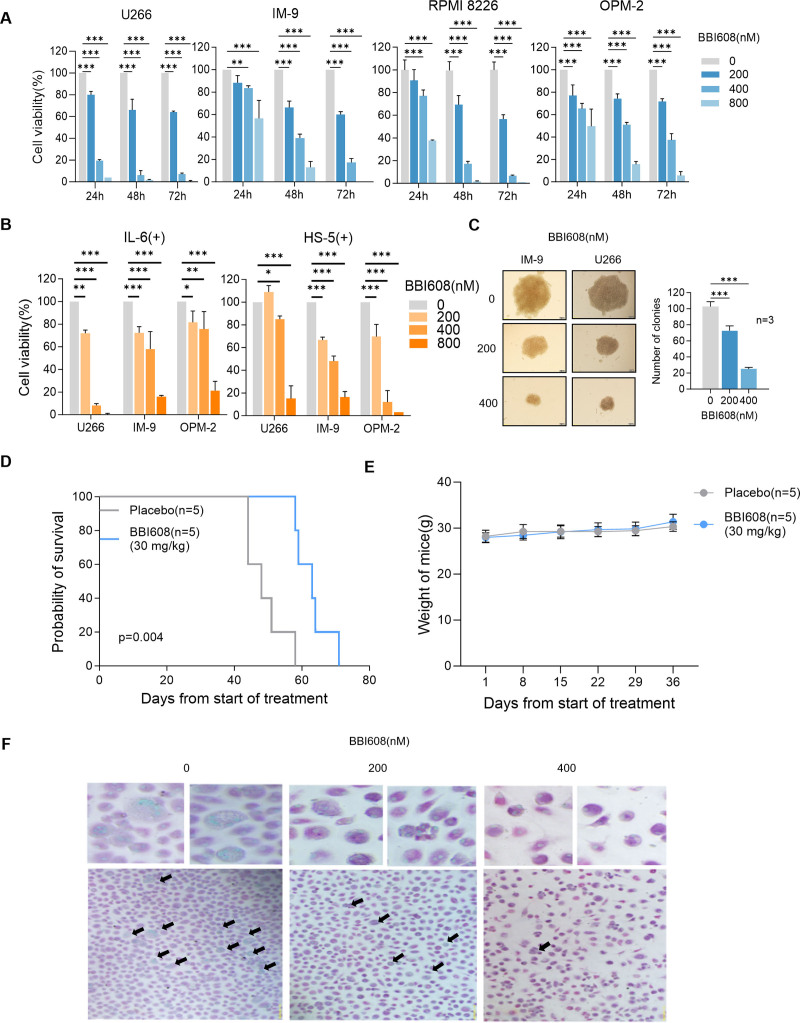
BBI608 blocked myeloma cell growth in vitro and in vivo. (A) A panel of MM cells (U266, IM-9, RPMI 8226, and OPM-2 cell lines) was cultured in RPMI-1640 medium, and treated with different concentrations of BBI608 (200, 400, and 800 nM) for 24, 48, and 72 h. Cell viability was measured by CCK-8 assay (**p < 0.010, ***p < .001) (n = 3 independent experiments [indep. exps], 3 technical replicates [tech. reps]). (B) MM cells were cocultured in RPMI-1640 medium under different conditions: with or without stimulation by IL-6 (25 ng/mL) or HS-5 conditional medium. Subsequently, the cells were treated with various concentrations of BBI608 for 48 h. Cell viability was measured by CCK-8 assay (*p < 0.050, **p < 0.010, ***p < .001) (n = 3 indep. exps, 3 tech. reps). (C) U266 and IM-9 cells were treated with different concentrations of BBI608 for 14 d. Colony formation assay was performed to evaluate the clonogenic ability after BBI608 treatment. The representative images of U266 and IM-9 cells are shown (left), and the number of colonies is shown in bar graph (right) (***p < .001) (n = 3 indep. exps, 3 tech. reps). (D–E) NSG mice were subcutaneously inoculated with RPMI 8226 cells, after tumor establishment, the mice were intraperitoneally administered either placebo or BBI608 (30 mg/kg, 5 d per week, n = 5) for 4 wk. (D) The survival of mice was analyzed by Kaplan–Meier survival curves (HR, 4.015; 95% CI, 0.8511–18.94. **p < 0.010). (E) The graph shows the changes in the body weight of mice over time. (F) Mononuclear cells cultured with 25 ng/mL of M-CSF and 50 ng/mL of RANKL were treated with BBI608 for 2 wk. Then, the cells were fixed and stained for TRACP. The TRAP+ osteoclast contains 3 or more nuclei per cell, and each osteoclast formation assay was performed at least 3 times using PBMCs from different donors (n = 3 indep. exps, 3 tech. reps). CI = confidence interval, CSF = colony-stimulating factor, HR = hazard ratio, IL = interleukin, MM = multiple myeloma, PBMC = peripheral blood mononuclear cell, TRCAP = tartrate-resistant acid phosphatase.

CCK-8 assays were performed to assess the cytotoxic effects of BBI608 on human MM cells after 24, 48, and 72 hours of treatment. BBI608 significantly suppressed the viability of U266, IM-9, RPMI 8226, and OPM-2 cells in a time- and dose-dependent manner, compared with the control (**Fig. 1A**). The IC50 values of BBI608 against MM cells were <1 μM (Figure S1A, https://links.lww.com/BS/A171). The BM microenvironment plays a critical role in MM progression by providing cytokines, chemokines, and cell–cell interactions that support MM cell survival and proliferation. To evaluate the efficacy of BBI608 under conditions mimicking the BM microenvironment, MM cells were cultured with human IL-6 (25 ng/mL) and HS-5 cell culture supernatants. The results demonstrated that BBI608 retained its inhibitory effects on MM cells, even in the simulated BM microenvironment (**Figs. 1B** and S1B, https://links.lww.com/BS/A171). Colony formation assays revealed that BBI608 significantly inhibited the proliferation of U266 and IM-9 cells (**Fig. 1C**). We investigated the in vivo efficacy of BBI608 in a murine MM model. The results demonstrated that BBI608 significantly prolonged the survival of tumor-bearing mice but had no obvious effect on body weight (**Fig. 1D–E**). To explore the impact of BBI608 on osteoclastogenesis, human PBMCs were induced to differentiate into osteoclasts and treated with BBI608 for 2 weeks. The marked reduction in the number of osteoclasts indicated that BBI608 effectively inhibited osteoclast formation (**Fig. 1F**). Taken together, our findings indicated that BBI608 effectively inhibited myeloma cell growth both in vitro and in vivo, highlighting its potential as a therapeutic agent against MM.

### 3.2. BBI608 induced cell cycle arrest and apoptosis and inhibited STAT3 activation in myeloma cells

**Figure 2.**
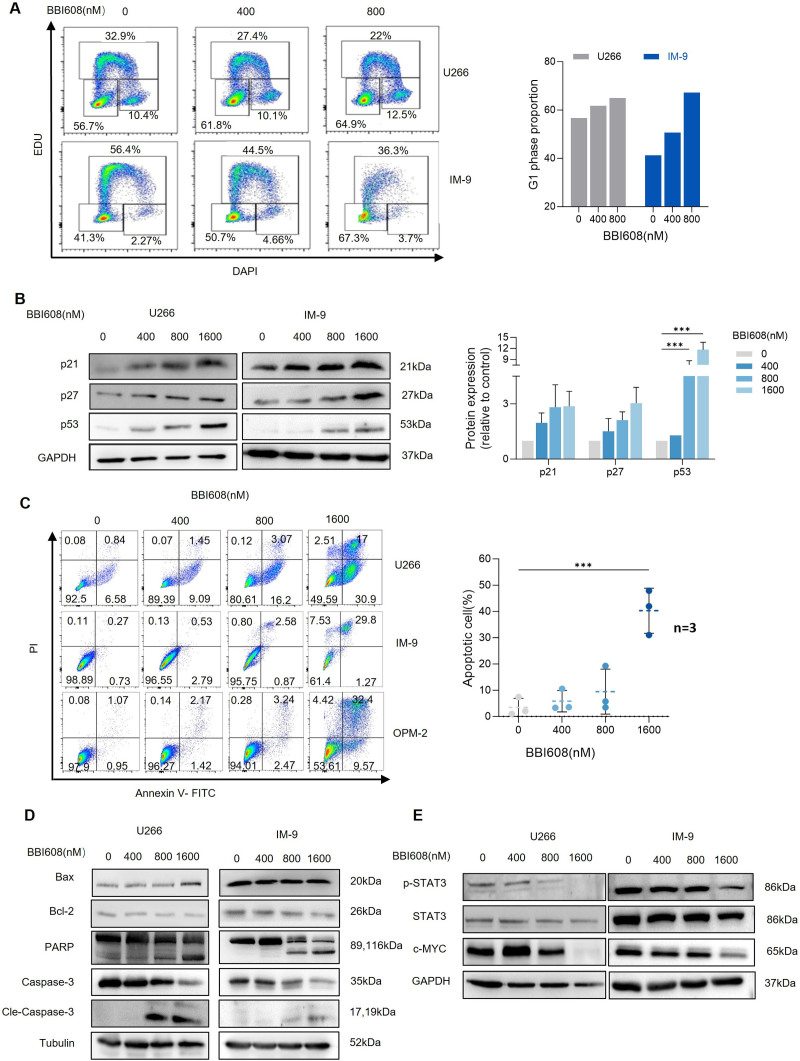
BBI608 induced cell cycle arrest, triggered apoptosis, and inhibited the activation of STAT3 of myeloma cells. (A) U266 and IM-9 cells were treated with BBI608 (400 and 800 nM) for 24 h. The cell cycle was evaluated by flow cytometric analysis after EdU incorporation. Percentages of cells in G1 phase were calculated and compared among the groups (n = 3 indep. exps). (B) U266 and IM-9 cells were treated with different concentrations of BBI608 (400, 800, and 1600 nM) for 24 h. WB assay was used to evaluate the expression of p21, p27, and p53 proteins. GAPDH was used as a loading control (***p < .001) (n = 3 indep. exps). (C) MM cells were treated with different concentrations of BBI608 (400, 800, and 1600 nM) for 24 h, and apoptotic cell death was analyzed by flow cytometric analysis staining with Annexin V/PI. The bar graph shows the ratio of apoptotic death cell (***p < .001) (n = 3 indep. exps). (D–E) U266 and IM-9 cells were treated with different concentrations of BBI608 for 24 h. (D) WB assay was used to evaluate the expression of Bax, Bcl-2, PARP, cleaved caspase-3 and caspase-3 proteins. β-tubulin was used as a loading control (n = 3 indep. exps). (E) WB assay was used to evaluate the expression of p-STAT3, STAT3, and c-MYC proteins. GAPDH was used as a loading control (n = 3 indep. exps). MM = multiple myeloma, PI = propidium iodide, STAT3 = signal transducer and activator of transcription 3.

To investigate the mechanism by which BBI608 inhibited MM cell proliferation, we examined its effects on cell cycle progression. Flow cytometric analysis revealed that BBI608 treatment significantly increased the proportion of cells in the G1 phase (**Fig. 2A**), concomitant with a marked decrease in S-phase populations, compared to untreated controls. Western blot analysis demonstrated the dose-dependent upregulation of the cyclin-dependent kinase inhibitors p21 and p27 as well as the tumor suppressor p53 in both cell lines following BBI608 exposure (**Fig. 2B**). The annexin V-FITC/PI staining assay indicated that BBI608 triggered significant apoptosis in U266, IM-9, and OPM-2 cells (**Fig. 2C**). Western blotting confirmed activation of the intrinsic apoptotic pathway. After treatment with BBI608, the protein levels of proapoptotic Bax, PARP, and cleaved caspase-3 increased, whereas the expression of the antiapoptotic protein Bcl-2 decreased. Therefore, the Bax/Bcl-2 ratio in the BBI608 treatment group increased significantly (**Fig. 2D**). As a selective STAT3 inhibitor, BBI608 downregulated the protein expression of STAT3 and c-Myc and the phosphorylation of STAT3 in U266 and IM-9 cells (**Fig. 2E**). These results indicated that BBI608 effectively blocked the G1/S transition, induced intrinsic apoptosis, and inhibited STAT3 activation in MM cells.

### 3.3. BBI608 eliminated myeloma stem-like cells

**Figure 3.**
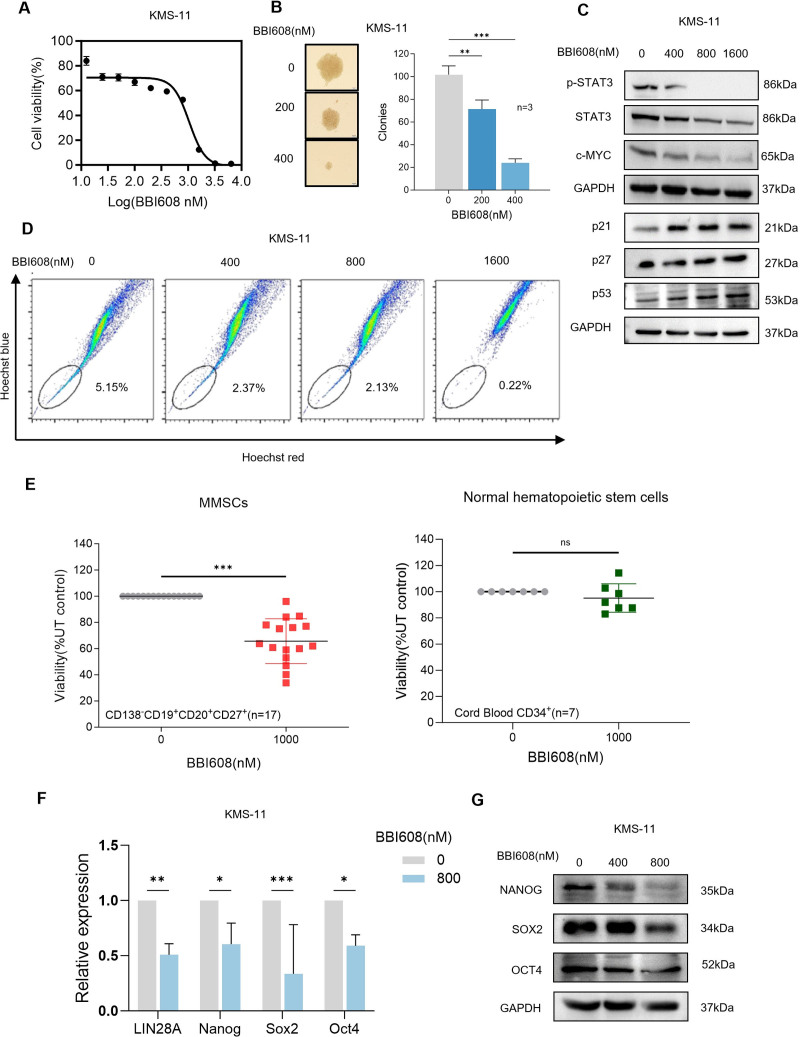
BBI608 eliminated myeloma stem-like cells. (A) KMS-11 cells were cultured in RPMI-1640 medium and treated with BBI608 for 72 h. The cell viability was measured by CCK-8 assay (n = 3 indep. exps, 3 tech. reps). (B) KMS-11 cells were treated with BBI608 (200 and 400 nM) for 14 d. The colony formation assay was performed to evaluate the clonogenic ability after BBI608 treatment. The representative image of KMS-11 cells is shown (left), and the number of colonies is shown in bar graph (right) (**p < 0.010, ***p < .001) (n = 3 indep. exps, 3 tech. reps). (C–D) KMS-11 cells were treated with different concentrations of BBI608 (400, 800, and 1600 nM) for 24 h. (C) WB assay was used to evaluate the expression of p21, p27, p53, p-STAT3, STAT3, and c-MYC proteins. GAPDH was used as a loading control (n = 3 indep. exps). (D) KMS-11 cells were stained with Hoechst 33342, analyzed by flow cytometry (n = 3 indep. exps). (E) Mononuclear cells isolated from peripheral blood of MM patients (left, n = 17) or umbilical cord blood of healthy individuals (right, n = 7) were treated with 1000 nM BBI608 for 24 h, stained with indicated antibodies and analyzed with flow cytometry (***p < .001). (F–G) KMS-11 cells were treated with different concentrations of BBI608 (400 and 800 nM) for 24 h. (F) Subsequently, the mRNA expression levels of stemness-related genes (*LIN28A*, *Nanog*, *Sox2*, and *Oct4*) were quantitatively analyzed using RT-qPCR (*p < 0.050, **p < 0.010, ***p < .001) (n = 3 indep. exps, 3 tech. reps). (G) WB assay was used to evaluate the expression of stemness-related genes (*Nanog*, *Sox2*, and *Oct4*) proteins (n = 3 indep. exps). MM = multiple myeloma, RT-qPCR = reverse transcription-quantitative polymerase chain reaction, STAT3 = signal transducer and activator of transcription 3.

Given the established role of STAT3 in regulating myeloma and tumor stem cells, we investigated the therapeutic potential of BBI608 targeting MMSCs using the KMS-11 myeloma cell line, which is enriched in cancer stem-like cells.^3,16^ CCK-8 assays were performed to assess the cytotoxic effects of BBI608 on KMS-11 cells after 72 hours of treatment. BBI608 significantly suppressed the viability of KMS-11 cells in a dose-dependent manner, compared to the control (**Fig. 3A**). Colony formation assays revealed that BBI608 significantly inhibited KMS-11 cell proliferation (**Fig. 3B**). Cell cycle analysis showed that treatment with BBI608 induced cell cycle arrest in KMS-11 cells, which was consistent with our observations in U266 and IM-9 cell lines. (**Fig. 3C**). To evaluate the effect of the drug on the stem cell population, we conducted a Hoechst 33342 exclusion test on KMS-11 cells treated with gradually increasing concentrations of BBI608 (400–1600 nM). Flow cytometric analysis revealed that the SP portion decreased in a dose-dependent manner (**Fig. 3D**), suggesting that BBI608 preferentially targeted stem cell-like cells.

Next, we extended our investigation to primary cells isolated from BM aspirates from 17 patients with MM and umbilical cord blood from 7 healthy donors. BBI608 treatment significantly reduced the viability of putative MMSCs (CD138^-^CD19^+^CD20^+^CD27^+^ population), with survival rates of 65.67% ± 5.45% when exposed to 1000 nM (**Fig. 3E**). Notably, the drug exhibited significantly less toxicity toward normal hematopoietic stem cells (CD34+), which maintained survival rates of 95.11% ± 5.92% at corresponding concentrations (*p* < 0.010). Given the central role of pluripotency factors in the maintenance of cancer stemness, we examined the effect of BBI608 on key stemness regulators. RT-qPCR and western blot analyses showed that KMS-11 cells treated with BBI608 had significantly downregulated mRNA expressions of Lin-28 homolog A (LIN28A), NANOG, SRY-related HMG box 2 (SOX2), and octamer-binding transcription factor 4 (OCT4) (**Fig. 3F and G**), suggesting disruption of the stem cell transcriptional program.

### 3.4. BBI608 triggered UPR and enhanced anti-myeloma immunity through ICD induction

**Figure 4.**
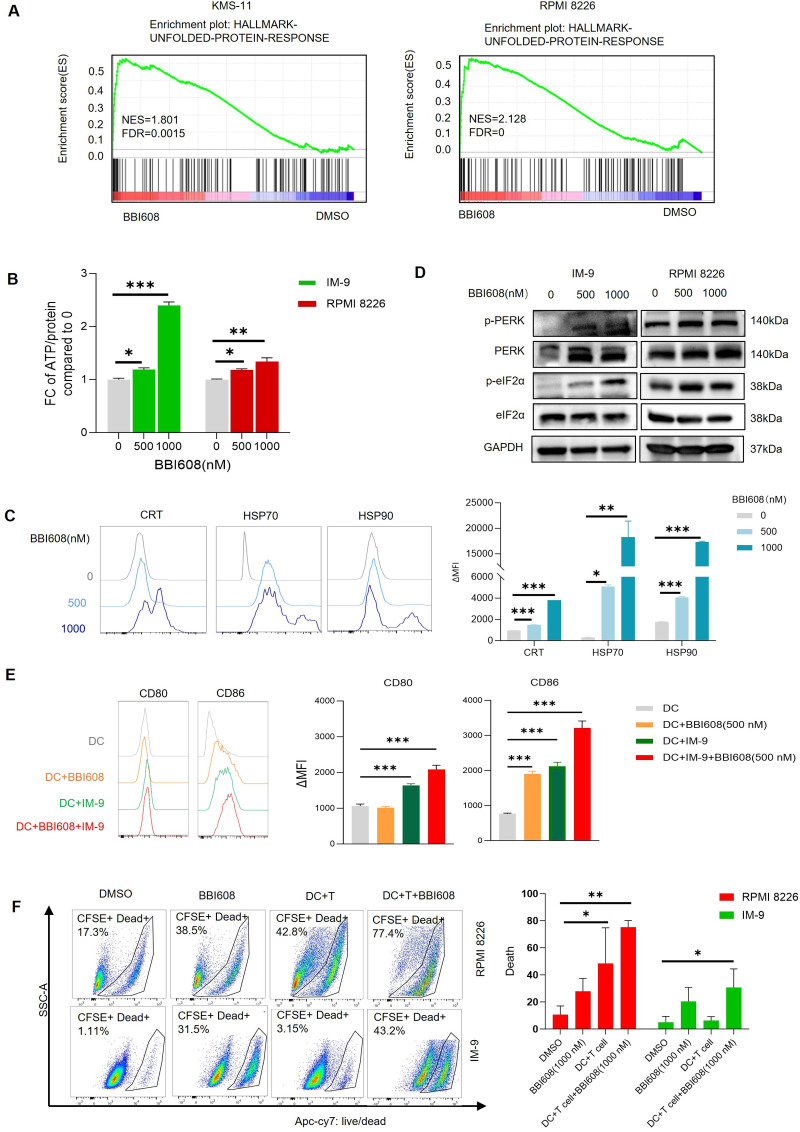
BBI608 triggered unfolded protein response and enhanced anti-myeloma immunity through immunogenic cell death induction. (A) KMS-11 and RPMI 8226 cells were treated with different concentrations of BBI608 for 24 h, and then subjected to RNA sequencing and GSEA. Enrichment plots of GSEA revealing significantly positive enrichments for the HALLMARK-UNFOLDED-PROTEIN-RESPONSE pathway. (B) IM-9 and RPMI 8226 cells were treated with BBI608 for 24 h. Extracellular ATP levels were measured using ATP assay kit (*p < 0.050, **p < 0.010, ***p < .001) (n = 3 indep. exps, 3 tech. reps). (C) IM-9 cells were treated with different concentrations of BBI608. The protein level of CRT, HSP70, and HSP90 was measured by flow cytometric analysis (left), the bar graph shows the ΔMFI of proteins (right) (n = 3 indep. exps). (D) IM-9 and RPMI 8226 cells were treated with different concentrations of BBI608 for 24 h. WB assay was used to evaluate the expression of PERK, p-PERK, eIF2α, and p-eIF2α proteins. GAPDH was used as a loading control (n = 3 indep. exps). (E) DCs were cocultured with RPMI 8226 and IM-9 cells pretreated with DMSO or BBI608 for 48 h, and the expression of CD80 and CD86 was detected by flow cytometry analysis (left). The bar graph shows the MFI of CD80 or CD86 (right) (n = 3 indep. exps). (F) The ratio of dead cells of the following groups was determined by live/dead staining: RPMI 8226/IM-9, 1000 nM BBI608 pretreated RPMI 8226/IM-9, RPMI 8226/IM-9+DCs+CD8+T cells, and 1000 nM BBI608 pretreated RPMI 8226/IM-9+DCs+CD8+T cells. The representative scatter plots are shown (n = 3 indep. exps). GSEA = gene set enrichment analysis, MFI = mean fluorescence intensity.

**Figure 5.**
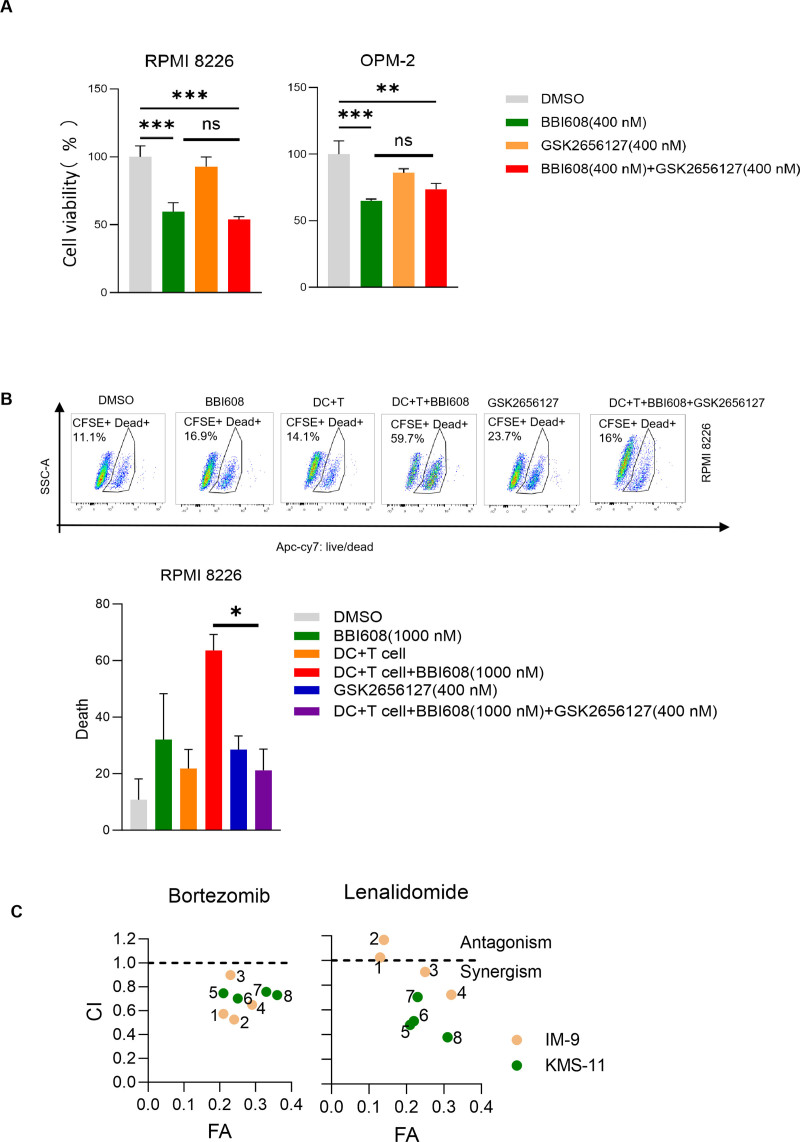
GSK2656127 weakened anti-myeloma immunity through inhibiting immunogenic cell death. (A) The ratio of live cells of the following groups: RPMI 8226/OPM-2. 400 nM BBI608 pretreated RPMI 8226/OPM-2, 400 nM GSK2656127 pretreated RPMI 8226/OPM-2, 400 nM BBI608 and 400 nM GSK2656127 pretreated RPMI 8226/OPM-2 (n = 3 indep. exps, 3 tech. reps) (**p < 0.010, ***p < .001). (B) The ratio of dead cells of the following groups was determined by live/dead staining: RPMI 8226, 1000 nM BBI608 pretreated RPMI 8226, RPMI 8226+DCs+CD8+T cells, 1000 nM BBI608 pretreated RPMI 8226+DCs+CD8+T cells, 400 nM GSK2656127 pretreated RPMI 8226, 1000 nM BBI608, and 400 nM GSK2656127 pretreated RPMI 8226+DCs+CD8+T cells. The representative scatter plots are shown (n = 3 indep. exps) (*p < 0.050). (C) IM-9 and KMS-11 cells were treated with BBI608 and bortezomib/lenalidomide for 48 h. The data were analyzed with CompuSyn software (n = 3 indep. exps, 3 tech. reps).

To further investigate the regulatory role of BBI608 in gene expression, RNA sequencing analysis was conducted on KMS-11 and RPMI 8226 cells, comparing BBI608-treated and untreated groups. GSEA and transcriptomic profiling of BBI608-treated KMS-11 and RPMI 8226 cells (24 hours with 500 and 1000 nM, respectively) revealed significant activation of the UPR pathway (**Fig. 4A**). Given the established link between ER stress and ICD, we characterized key ICD markers in treated MM cells.^17–20^ BBI608 significantly increased intracellular ATP levels (2.1- and 1.8-fold in IM-9 and RPMI 8226 cells, respectively (**Fig. 4B**) and enhanced the surface exposure of CRT (3.2-fold), HSP70 (2.7-fold), and HSP90 (2.1-fold) in IM-9 cells (**Fig. 4C**). Mechanistically, BBI608 treatment activated the PERK-eIF2α ER stress axis, as evidenced by increased phosphorylation of both PERK (2.3- and 2.1-fold) and eIF2α (1.9- and 1.7-fold) in IM-9 and RPMI 8226 cells, respectively (**Fig. 4D**). The ICD-inducing capacity translated into enhanced DC maturation, with BBI608-pretreated MM cells showing increased DC expression of CD86 (3.1-fold) and CD80 (2.4-fold) (**Fig. 4E**). Functional co-culture assays demonstrated that BBI608-primed MM cells significantly boosted DC-mediated CD8+ T cell cytotoxicity (**Fig. 4F**). Compared with the use of BBI608 alone, the combination of BBI608 with the PERK inhibitor (GSK2656127) resulted in no significant change in MM cell proliferation (**Fig. 5A**). However, the DC-mediated cytotoxicity of CD8+ T cells was significantly reduced (**Fig. 5B**). The results suggest that BBI608-induced ICD is indeed partly mediated through the PERK-eIF2α ER stress axis. Furthermore, BBI608 showed synergistic anti-myeloma effects with bortezomib and lenalidomide, as determined by the Chou-Talalay analysis (**Figs. 5C** and S1C, https://links.lww.com/BS/A171, Tables 1 and 2). Our findings established that BBI608 induced ER stress–mediated ICD in myeloma cells, promoting anti-tumor immunity through DC activation and enhanced T cell responses while exhibiting favorable combinatory potential with proteasome inhibitors and immunomodulatory drugs (ImiDs) in vitro.

**Table 1:** BBI608 has a combination effect with bortezomib.

No.	Cell	BTZ (nM)	BBI608 (nM)	Effect	CI
1	IM-9	0.125	50	0.21	0.57
2	IM-9	0.25	50	0.24	0.52
3	IM-9	0.125	100	0.23	0.89
4	IM-9	0.25	100	0.29	0.64
5	KMS-11	0.125	50	0.21	0.74
6	KMS-11	0.25	50	0.25	0.70
7	KMS-11	0.125	100	0.33	0.75
8	KMS-11	0.25	100	0.36	0.73

CI = combination index.

IM-9 and KMS-11 cells were cocultured with varying concentrations of BBI608 (50 and 100 nM) and bortezomib (0.125 and 0.25 nM)/lenalidomide (2.5 and 5 μM) for 48 h. Cell viability was measured by CCK-8 assay at 48 h, and the CI was calculated using Compusyn software.

**Table 2:** BBI608 has a combination effect with lenalidomide.

No.	Cell	LEN (μM)	BBI608 (nM)	Effect	CI
1	IM-9	2.5	50	0.13	1.02
2	IM-9	5	50	0.14	1.16
3	IM-9	2.5	100	0.25	0.90
4	IM-9	5	100	0.32	0.72
5	KMS-11	2.5	50	0.21	0.47
6	KMS-11	5	50	0.22	0.50
7	KMS-11	2.5	100	0.23	0.70
8	KMS-11	5	100	0.31	0.37

CI = combination index.

IM-9 and KMS-11 cells were cocultured with varying concentrations of BBI608 (50 and 100 nM) and bortezomib (0.125 and 0.25 nM)/lenalidomide (2.5 and 5 μM) for 48 h. Cell viability was measured by CCK-8 assay at 48 h, and the CI was calculated using Compusyn software.

## 4. DISCUSSION

**Figure 6.**
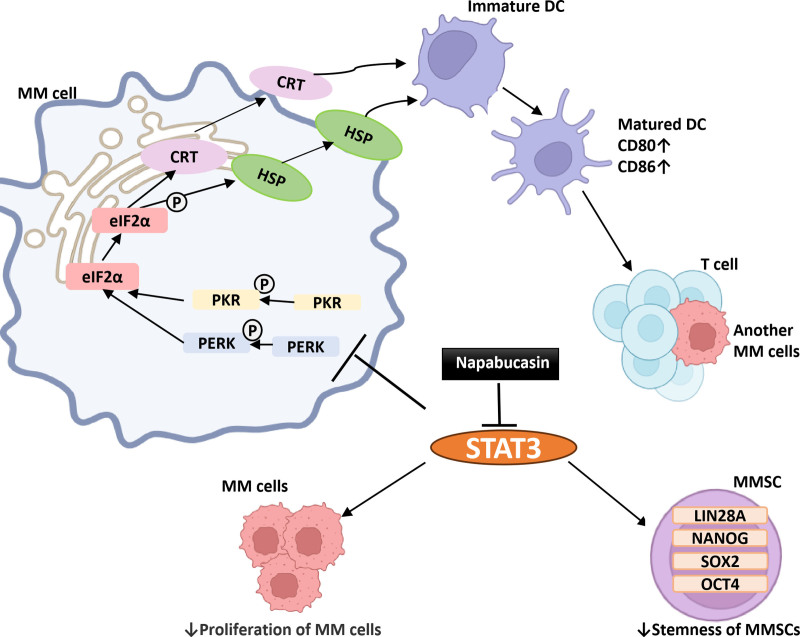
Schematic illustration of STAT3 targeting suppressing multiple myeloma proliferation and stem cell activity while promoting ICD induction. CRT = calreticulin, DC = dendritic cell, HSP = heat shock protein, ICD = immunogenic cell death, MMSC = multiple myeloma stem-like cell, PERK = protein kinase R-like endoplasmic reticulum kinase, PKR = protein Kinase R, STAT3 = signal transducer and activator of transcription 3.

MM is a heterogeneous disease characterized by transcriptional activation, metabolic dysregulation, drug resistance, and other hallmarks of aggressive disease.^2,21,22^ Constitutively active STAT3 is expressed in the majority of CD138+ primary MM cells and plays a crucial role in maintaining MM survival and proliferation, regulating the tumor microenvironment, and inducing immunosuppression. Therefore, targeting STAT3 is a promising therapeutic strategy. BBI608 is an orally bioavailable small-molecule inhibitor that targets the STAT3 pathway and has reported activity against CSCs.^23,24^ In this study, we evaluated the anti-myeloma effects of BBI608 both in vitro and in vivo. Our findings demonstrated that BBI608 exhibits potent cytotoxicity in MM by suppressing the proliferation of both bulk myeloma cells and stem-like subpopulations. Furthermore, BBI608 induced ICD, suggesting a dual mechanism of action involving direct tumor cell killing and enhanced anti-myeloma immunity (**Fig. 6**). Notably, while the effective doses of BBI608 in MM were comparable to those used in solid tumors, MM cells exhibit heightened sensitivity to STAT3 inhibition.^12,25,26^ This dependence on persistent STAT3 signaling may allow BBI608 to achieve therapeutic efficacy at lower concentrations, highlighting its potential as a targeted strategy for MM.

Despite significant advancements in the systemic treatment of MM over the past 2 decades, including proteasome inhibitors, ImiDs, autologous stem cell transplantation (ASCT), and chimeric antigen receptor T-cell (CAR-T) therapy, improvements in survival rates remain inconsistent across patient populations.^27^ Drug resistance and relapse have become increasingly prominent clinical challenges, which may be partly attributed to the persistence of stem cell-like subpopulations within MM cells.^28^ CSCs in MM express key pluripotency-associated transcription factors such as LIN28, NANOG, SOX2, OCT4, and c-MYC, which contribute to their self-renewal and therapy-resistant properties. Additionally, intracellular signaling pathways, including STAT3, play a crucial role in regulating CSC phenotypes.^29^ SP cells are characterized by their ability to efflux Hoechst 33342, which results in low fluorescence staining and a distinct flow cytometry profile, and exhibit stem-like properties similar to those of hematopoietic stem cells.^30^ In this study, we demonstrated that BBI608 significantly reduced the SP fraction in KMS-11 MM cells and downregulated critical stemness factors (LIN28A, NANOG, SOX2, and OCT4) that are essential for maintaining pluripotency. Notably, BBI608 exhibited selective cytotoxicity, with a stronger inhibitory effect on MM stem cells than on normal umbilical cord blood hematopoietic stem cells. These findings suggest that BBI608 has considerable therapeutic potential in targeting MMSCs, offering a promising strategy for overcoming drug resistance and relapse.

Regulatory cell death (RCD) is a genetically programmed form of cell death that initiates inflammatory responses when induced by cellular stress. This process ultimately promotes cytotoxic T lymphocyte (CTL)–driven adaptive immunity and establishes long-term immune memory.^30^ ICD, a subset of RCD, primarily activates anti-tumor immune responses through the release of DAMPs, making it an appealing strategy for cancer immunotherapy.^31^ Given its immunomodulatory effects, ICD has emerged as an attractive strategy for cancer immunotherapy.^32^ Apoptosis has long been considered an immunologically silent mechanism in RCD. Nevertheless, apoptosis induced by specific stimuli, such as anthracycline-based chemotherapeutic agents and oxidative stress, can be converted into ICD upon exposure to CALR and the release of ATP, high-mobility group box 1 protein (HMGB1), and other DAMPs, thereby triggering adaptive immunity mediated by CD8+ cytotoxic T lymphocytes. Notably, the immunogenicity of dying cells is not determined by their morphological features but rather by the specific DAMPs that are released or exposed in a well-orchestrated spatiotemporal sequence. Collectively, these characteristics define a distinct subtype of regulated cell death, namely ICD.^30,33^

For example, bortezomib induces ER stress and the UPR by inhibiting the 26S proteasome, which not only triggers apoptosis of MM cells but also elicits ICD through a series of hallmark events, including CALR exposure, ATP release, and HMGB1 secretion. Moreover, the activation of the cGAS/STING pathway and type I interferon (IFN) response serves as a critical hub linking apoptosis to ICD and mediating anti-tumor immunity, and its clinical efficacy is highly dependent on CALR-driven host anti-tumor immunity.^32^ However, identifying potent ICD inducers and advancing ICD-based therapies from preclinical research to clinical applications remain critical challenges. In this study, DCs cocultured with IM-9 cells pretreated with BBI608 expressed high levels of CD86 and CD80 on their cell surfaces, indicating that BBI608 triggered ICD and activated DC-mediated immune responses. This suggests enhanced DC maturation and activation of downstream immune responses. Furthermore, cytotoxicity assays revealed that BBI608 pretreatment significantly enhanced the tumor-killing capacity of CD8+ T cells in vitro, supporting its role in promoting anti-MM immunity. Immunotherapy has revolutionized oncology by enabling targeted tumor cytotoxicity and the induction of immune memory, thereby reducing the risk of relapse. Notably, bortezomib and lenalidomide, the cornerstone therapies for MM, are known ICD inducers that activate CD8+ T cells, enhance DC-mediated antigen uptake, and improve antigen presentation.^34^ Our findings suggest that combining BBI608 with these agents can synergistically enhance the therapeutic efficacy. However, further investigation is needed to determine whether BBI608 modulates other immune components such as regulatory T cells (Tregs) and whether it remodels the tumor microenvironment to suppress MM progression.

In summary, our study demonstrated that BBI608 exhibited multifaceted anti-myeloma effects, including direct cytotoxicity against MM cells, suppression of MM stem cell stemness, induction of ER stress, and promotion of ICD-mediated immune clearance. These findings have established BBI608 as a promising therapeutic candidate for multiple myeloma, particularly when combined with existing ICD-inducing regimens.

## ACKNOWLEDGMENTS

This work was supported by the National Natural Science Foundation of China (82570258 and 82500273), Natural Science Foundation of Jiangsu Province (BK20250157), and the Qinglan Project of China to YY and the Key Project of Jiangsu Provincial Health Commission (K2024066).

We thank the Key Laboratory of Bone Marrow Stem Cell of XuZhou Medical University for technical support.

## ETHICAL APPROVAL

Approval of the research protocol by an Institutional Review Board: This study has been approved by the Hospital Medical Ethics Committee of the Affiliated Hospital of Xuzhou Medical University, and conformed to the standards set by the Declaration of Helsinki. Animal Studies: All the animal experiments were approved by the Animal Research Committee of Xuzhou Medical University and conducted in accordance with institutional guidelines for animal care and handling.

## AUTHOR CONTRIBUTIONS

Y.Y. designed the experiments, wrote the manuscript, and prepared the presentation of the data. P.Y. helped analyze the data and revised the manuscript. Y.S., Y.Z., and W.L. performed the experiments, analyzed the results, and created the figures. M.Y., W.W., T.L., and J.G. helped with the experiments. T.L. helped with the data analysis. H.C. and M.N. oversaw the interpretation of data.

## Supplementary Material
