# Supplementary material for: STAT3 inhibition eradicates multiple myeloma stem-like cells and induces immunogenic cell death
Source: Blood Sci. 2026 Jul 31;8(3):e00301. doi: 10.1097/BS9.0000000000000301 (PMC13441156; doi:10.1097/BS9.0000000000000301)
Supplement: Supplementary file 1 [file bs9-8-e00301-s001.pdf]

SFigure 1

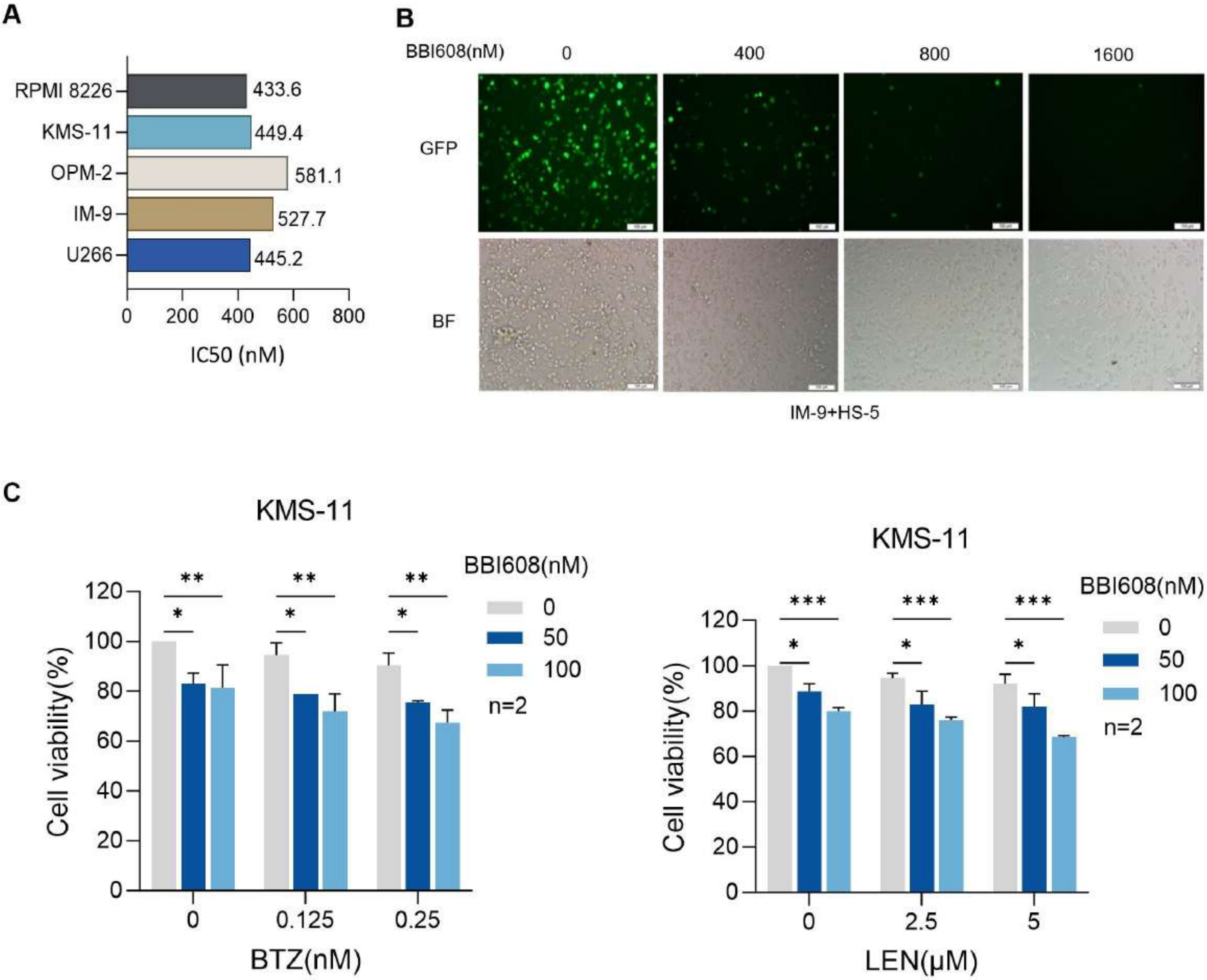
A
RPMI 8226
KMS-11
OPM-2
IM-9
U266
433.6
449.4
581.1
527.7
445.2
0
200
400
600
800
IC50 (nM)
B
BBI608(nM)
0
400
800
1600
GFP
BF
IM-9+HS-5
C
KMS-11
BBI608(nM)
0
50
100
n=2
Cell viability(%)
120
100
80
60
40
20
0
0.125
0.25
BTZ(nM)
KMS-11
BBI608(nM)
0
50
100
n=2
Cell viability(%)
2.5
5
LEN(μM)
